# Current use of the pelvic organ prolapse quantification system in clinical practice among Korean obstetrician-gynecologists

**DOI:** 10.1186/s12905-021-01354-w

**Published:** 2021-05-18

**Authors:** Soo Rim Kim, Dong Hoon Suh, Myung Jae Jeon

**Affiliations:** 1 Obstetrics and Gynecology, International St. Mary’s Hospital, Catholic Kwandong University College of Medicine, Incheon, Republic of Korea; 2 Obstetrics and Gynecology, Seoul National University Bundang Hospital, Seongnam, Republic of Korea; 3 Department of Obstetrics and Gynecology, Seoul National University College of Medicine, Seoul National University Hospital, 101 Daehak-ro, Jongno-gu, Seoul, 03080 Republic of Korea

**Keywords:** Evaluation, Pelvic organ prolapse, Pelvic organ prolapse quantification system, Surgical decision-making

## Abstract

**Background:**

To assess current use of the Pelvic Organ Prolapse Quantification (POP-Q) system in clinical practice among Korean obstetrician-gynecologists.

**Methods:**

A web-based questionnaire was sent to 780 Korean Society of Obstetrics and Gynecology members. The items evaluated in the questionnaire were demographic characteristics and current use of the POP-Q system in the evaluation of pelvic organ prolapse (POP) and surgical decision-making. Differences between POP-Q users and nonusers were analyzed by using the two-sample t-test and chi-squared test.

**Results:**

One hundred twenty-six members (16%) responded to the survey. Of the respondents, 48% reported using the POP-Q system in the evaluation of POP. Members who were female, urogynecologists, or performed a high volume of prolapse surgery were more likely to use the POP-Q system (p < 0.05). All but one of the POP-Q users reported using the specific criteria to determine whether each compartmental prolapse should be corrected during prolapse surgery. Most respondents used stage 2 or the hymen as a threshold for prolapse to be corrected for all compartments.

**Conclusions:**

Less than half of Korean obstetrician-gynecologists use the POP-Q system in the evaluation of POP. Almost all of POP-Q users make a surgical decision based on the results of the POP-Q examination.

**Supplementary Information:**

The online version contains supplementary material available at 10.1186/s12905-021-01354-w.

## Background

Pelvic organ prolapse (POP) is the downward descent of the pelvic organs that results in protrusion of the vagina, uterus, or both. It affects almost half of all women older than 50 years of age, and often causes bladder, bowel, and pelvic symptoms that can have an adverse effect on a woman’s daily activities and quality of life [1, 2]. Epidemiologic studies have shown that 11–19% of women undergo operation for POP during their lifetime [3, 4].

POP is diagnosed during a pelvic examination. Although several grading/staging systems have been developed to document the extent of prolapse, the most common system with international acceptance is the Pelvic Organ Prolapse Quantification (POP-Q) system. This system involves quantitative measurements of six vaginal points representing anterior, apical, and posterior vaginal prolapse in centimeters relative to the hymen, and has been shown to have good interobserver and intraobserver reliabilities [5, 6]. Nonetheless, it does not identify the underlying support defects, which may limit its use in clinical practice including surgical decision-making.

There are few reports on the use of the POP-Q system in clinical practice. Surveys of American Urogynecologic Society (AUGS) and International Continence Society (ICS) members in 2004 and 2011 showed that the POP-Q is not widely used in their clinical practice despite its endorsement by relevant professional societies. The main reason for not using the POP-Q was the lack of perceived clinical relevance compared to the time and effort involved in its used [7, 8].

The aim of this study was to assess current use of the POP-Q system for the evaluation of POP and surgical decision-making among Korean obstetrician-gynecologists.

## Method

### Study design and participants

After obtaining permission from the Korean Society of Obstetrics and Gynecology (KSOG), a web-based questionnaire was sent to 780 members of this society in September 2019. The questionnaire developed for this study is provided as Additional File 1. To increase the response rate to the survey, a follow-up email was sent within a 1-month interval asking members to respond to the survey; surveys were carefully screened to avoid duplicate participation.

### Data collection

The items evaluated in the questionnaire were demographic characteristics including age, sex, subspecialty, fellowship training for prolapse surgery, and surgical experience and volume; and current use of the POP-Q system in clinical practice. Nonusers were asked to indicate what other quantification system they do use, if applicable. POP-Q users were asked to describe the manner in which they perform the POP-Q measurements, specifically in relation to the patient’s position and bladder volume, and if they repeat the POP-Q examination with simulated apical support. POP-Q users were also asked if they use the POP-Q measurements in surgical decision-making, which degree of prolapse for each compartment they think is to be corrected, and if they perform separate repair for anterior or posterior prolapse resolved under simulated apical support.

### Statistical analysis

Data were analyzed using SPSS software (version 22; IBM Corp., Armonk, NY, USA). A comparison of continuous and categorical variables between POP-Q users and nonusers was performed using the two-sample t-test and chi-squared test, respectively. A p value < 0.05 was considered statistically significant.

## Results

One hundred twenty-six members (16%) responded to the survey. Table 1 shows the demographics of the survey respondents. Most respondents were male (68%) and had a subspecialty other than urogynecology (76%). Of the respondents, 16% completed a fellowship for prolapse surgery, 52% had more than 10 years of experience, and 14% conducted more than 50 cases of prolapse surgery per year.

**Table 1:** Respondents’ demographics (n = 126)

Variable	Value
Age, year	47.8 ± 9.3
Sex	
Male	86 (68.3)
Female	40 (31.7)
Subspecialty	
Urogynecology	30 (23.8)
Others	96 (76.2)
Fellowship training for prolapse surgery	
No	106 (82.2)
Yes	20 (15.5)
Years of experience in clinical practice	
* ≤5*	34 (27.0)
6–10	27 (21.4)
11–15	26 (20.6)
16–20	20 (15.9)
> 20	19 (15.1)
Surgical volume, number of cases/year	
*≤*20	74 (58.7)
21–50	35 (27.8)
51–100	12 (9.5)
> 100	5 (4.0)

Values are presented as mean ± standard deviation or number (%)

Sixty-one (48%) respondents reported using the POP-Q system in their clinical practice. Of the 65 respondents who did not use the POP-Q, 63 used the Baden-Walker system and two did not use any quantification system. Members who were female, urogynecologists, or performed a high volume of prolapse surgery were more likely to use the POP-Q system (Table 2).

**Table 2:** Comparison of characteristics between POP-Q users and nonusers

Variable	POP-Q users (n = 61)	Nonusers (n = 65)	P value*
Age, year	48.1 ± 9.5	47.6 ± 9.2	0.765
Sex			0.031
Male	36 (59.0)	50 (76.9)	
Female	25 (41.0)	15 (23.1)	
Subspecialty			0.006
Urogynecology	21 (34.4)	9 (13.8)	
Others	40 (65.6)	56 (86.2)	
Fellowship for prolapse surgery			0.524
No	50 (82.0)	56 (86.2)	
Yes	11 (18.0)	9 (13.8)	
Years of experience in clinical practice			0.966
*≤*5	18 (29.5)	16 (24.6)	
6–10	12 (19.7)	15 (23.1)	
11–15	13 (21.3)	13 (20.0)	
16–20	9 (14.8)	11 (16.9)	
> 20	9 (14.8)	10 (15.4)	
Surgical volume, number of cases/year			0.009
*≤ *20	35 (57.4)	39 (60.0)	
21–50	12 (19.7)	23 (35.4)	
51–100	9 (14.8)	3 (4.6)	
> 100	5 (8.2)	0	

Values are presented as mean ± standard deviation or number (%)

POP-Q, Pelvic Organ Prolapse Quantification

^*^Calculated from the two-sample t-test for continuous variables or chi-squared test for categorical variables

Table 3 shows details of the POP-Q examination. Forty-four percent of the POP-Q users performed the examination with patients in the 45-degree upright sitting position, 31% in the supine position, and 20% in the standing position to evaluate prolapse. Most (61%) POP-Q users examined patients with an empty bladder. Thirty-three percent of the POP-Q users repeated the POP-Q examination with simulated apical support.

**Table 3:** Details of the POP-Q examination (n = 61)

Variable	Value
Position	
Supine	19 (31.1)
45°-upright sitting	27 (44.3)
Standing	12 (19.7)
Others	3 (4.9)
Bladder volume	
Empty	37 (60.7)
Any volume	24 (39.3)
Repeat the POP-Q measurements with simulated apical support	
No	41 (67.2)
Yes	20 (32.8)

Values are presented as number (%)

*POP-Q* pelvic organ prolapse quantification

Table 4 shows the surgical decision-making pattern among the POP-Q users. All but one of the POP-Q users reported using the POP-Q measurements in surgical decision-making. They used various criteria to determine whether each compartmental prolapse should be corrected during prolapse surgery. Most respondents used stage 2 or the hymen as a threshold for prolapse to be corrected for all compartments. Among the respondents repeating the POP-Q examination with simulated apical support, only 45% reported that they did not perform separate repair for anterior or posterior prolapse resolved under simulated apical support.

**Table 4:** Surgical decision-making pattern among the POP-Q users (n = 61)

Variable	Value
Apical prolapse to be corrected	
Any (regardless of the degree of prolapse)	1 (1.6)
POPQ point C > − (TVL-2) (stage 1 or greater)	3 (4.9)
POPQ point C > − 1/2 × TVL	6 (9.8)
POPQ point C ≥ -1 (stage 2 or greater)	28 (45.9)
POPQ point C > 0 (beyond the hymen)	23 (37.7)
Anterior or posterior prolapse to be corrected	
Any (regardless of the degree of prolapse)	1 (1.6)
POPQ point Ba or Bp > − 3 (stage 1 or greater)	1 (1.6)
POPQ point Ba or Bp ≥ − 1 (stage 2 or greater)	31 (50.8)
POPQ point Ba or Bp > 0 (beyond the hymen)	28 (45.9)
Separate repair for anterior or posterior prolapse resolved under simulated apical support	
No	9/20* (45.0)
Yes	11/20* (55.0)

Values are presented as number (% among the total POPQ users) unless specified otherwise

*POPQ* pelvic organ prolapse quantification, *TVL* total vaginal length

^*^Respondents who repeated the POPQ measurements with simulated apical support were included in the nominator and denominator

## Discussion

This is the first study to assess the use of the POP-Q system in the evaluation of POP and surgical decision-making among Korean obstetrician-gynecologists. The results revealed that only 48% of the respondents used the POP-Q in their clinical practice. Almost all of the POP-Q users made a surgical decision for each compartmental prolapse on the basis of the POP-Q measurements. Most respondents used stage 2 or the hymen as a threshold for prolapse to be corrected during prolapse surgery for all compartments.

Our results are in line with the findings of previous studies conducted in AUGS and ICS members. The first survey in 2004 showed that only 40% of the respondents routinely used the POP-Q system and 20% sometimes used it [7]. The second survey in 2011 showed that 76% of the respondents were currently using the POP-Q. Of those respondents who reported current use of the POP-Q, 80% used it often in daily practice, 93% used it preoperatively, 66% used it postoperatively, and 84% used the POP-Q only when participating in research or clinical trials [8]. These results showed that the POP-Q system is not being routinely used in clinical practice despite improvement in the user rate. Compared with the recent survey of AUGS and ICS members [8], our survey showed a lower rate of POP-Q use. This may be explained by the difference in the study population. Because of the lack of urogynecologic subspecialists, a significant proportion of prolapse surgeries is performed by surgeons who have a subspecialty other than urogynecology in Korea. To understand the current use of the POP-Q system in Korea, our survey was conducted in all KSOG members, and this may have lowered the user rate. Among the urogynecologic subspecialists, 70% used the POP-Q system in their practice.

We also found that there is considerable variability in the technical performance among the POP-Q users. It is unclear whether these variations are problematic. However, our survey showed that almost all surgeons determine whether each compartmental prolapse should be corrected on the basis of the degree of prolapse. Patient position and bladder volume may affect the extent of prolapse [9, 10], and these factors should be standardized.

Despite criticism of the lack of clinical relevance by some AUGS and ICS members, our results showed that the POP-Q measurements are used in surgical decision-making. However, there is considerable variability in the specific criteria used to determine whether prolapse should be corrected. This finding is not surprising considering that there are no consensus statements or guidelines about such criteria [11]. Nonetheless, most respondents reported using stage 2 or the hymen as a threshold for prolapse to be corrected for all compartments. Unlike POP-Q stage 1 anterior or posterior prolapse, stage 1 apical prolapse is likely to result in prolapse symptoms [12, 13]. Recent studies suggested that specialists should consider an apical suspension procedure in patients with POP-Q point C ≥ − 3, ≥ − 5, or > − 1/2 × total vaginal length [12–14]. Adequate support for the vaginal apex is essential to ensure a durable surgical repair for POP, and surgical correction of the anterior and posterior walls may fail unless the apex is adequately supported [15, 16]. Standardized definitions and guidelines are needed for clinically significant apical prolapse and when an apical suspension procedure should be performed.

Lastly, we assessed how much simulated apical support was performed during the POP-Q examination. Although the degree of prolapse in each compartment can be assessed by the POP-Q examination, the impact of prolapse on one compartment of the vagina on another cannot be assessed. Several studies have demonstrated that a significant proportion of cases of anterior vaginal wall prolapse are related to apical vaginal prolapse [17–19]. Simulated apical support is a test to assess the degree of anterior or posterior vaginal wall prolapse while holding the apex at approximately the depth of the total vaginal length, and it may help determine the need of separate anterior or poster repair at the time of apical suspension [11, 20]. However, we found that this test is underused in the evaluation of POP and surgical decision-making among the POP-Q users. This may be related to the lack of a clinical study to support its clinical relevance and the limited use of an apical suspension procedure during pelvic reconstruction [21].

There are some limitations in our study. First, we could not directly contact members because of KSOG policy. Our survey was anonymous, and targeted reminders to non-respondents, which may have increased the response rate, could not be sent. Second, our survey findings may also be subjected to sampling bias. Most respondents were from a tertiary medical center; therefore, these results may be more representative of academic practices rather than general practices.

## Conclusion

In conclusion, our study showed that less than half of Korean obstetrician-gynecologists use the POP-Q system in the evaluation of POP. In addition, almost all of the POP-Q users make a surgical decision on the basis of the results of the POP-Q examination.

## Supplementary Information

**Additional file 1.** Survey questionnaire.

## Data Availability

The datasets generated and/or analyzed during the current study are not publicly available due KSOG policy but are available from the corresponding author on reasonable request.

## Abbreviations

AUGS: American Urogynecologic Society
ICS: International Continence Society
KSOG: Korean Society of Obstetrics and Gynecology
POP: Pelvic organ prolapse
POP-Q: Pelvic organ prolapse quantification
