# Supplementary material for: Current use of the pelvic organ prolapse quantification system in clinical practice among Korean obstetrician-gynecologists
Source: BMC Womens Health. 2021 May 18;21:207. doi: 10.1186/s12905-021-01354-w (PMC8130334; doi:10.1186/s12905-021-01354-w)
Supplement: Supplementary file 1 — Additional file 1. Survey questionnaire. [file 12905_2021_1354_MOESM1_ESM.docx]

**Part I. General information**

**1. How old are you?**

**2. What is your gender?**

□ Male □ Female

**3. What is your subspecialty?**

□ Maternal-Fetal Medicine □ Gynecologic Oncology □ Reproductive Endocrinology

□ Urogynecology □ None

**4. Did you take a separate course for fellowship for pelvic organ prolapse surgery?**

□ Yes □ No

**5. How long have you performed your own pelvic organ prolapse surgery?**

□ Less than 5 years □ 6-10 years □ 11-15 years □ 16-20 years □ More than 20 years

**6. How many prolapse surgeries do you perform per year?**

□ Less than 20 cases □ 21-50 cases □ 51-100 cases □ More than 100 cases

**Part II. Evaluation of Pelvic Organ Prolapse and Decision making for Pelvic Reconstruction**

**Do you evaluate pelvic organ prolapse using the POP-Q system?**

□ Yes □ No

- **If you answered “no”, how do you evaluate it?**

□ Baden-Walker Halfway Scoring system □ Others

- **If you answered “yes”,**

**1. What position does the patient take when she is examined?**

□ Supine □ 45-degree upright sitting □ Standing □ Others

**2. Do you examine the patient after she empties her bladder?**

□ Yes □ No (regardless of the bladder volume)

**3. Which degree of prolapse do you judge as a significant anterior or posterior vaginal wall prolapse that requires correction?**

□ All need to be corrected regardless of the degree of prolapse

□ Stage I or greater (POPQ point Ba or Bp > -3)

□ Stage II or greater (POPQ point Ba or Bp ≥ -1)

□ Beyond the hymen (POPQ point Ba or Bp >0)

**4. Which degree of prolapse do you judge as a significant apical prolapse that requires correction?**

□ All need to be corrected regardless of the degree of prolapse

□ Stage I or greater (POPQ point C > -(TVL-2))

□ Beyond the half-way point of vagina (POPQ point C > -1/2 x TVL)

□ Stage II or greater (POPQ point C ≥ -1)

□ Beyond the hymen (POPQ point C >0)

**5. Do you perform additional simulated apical support test* during the POP-Q examination?**

□ Yes □ No

*Simulated apical support test: It is evaluated the maximum degree of prolapse of the anterior and posterior vaginal wall while supporting the vaginal apex using the posterior blade of speculum or ring forceps

**-> If you answered “yes”,**

**5-1) Do you think that separate repair is required for anterior or posterior prolapse resolved under simulated apical support?**

□ Yes (required) □ No (not required)
